# Psychological Stress Is Associated with Increased Cancer Risk in Dogs

**DOI:** 10.3390/ani13111869

**Published:** 2023-06-03

**Authors:** Isain Zapata, Alexander W. Eyre, Carlos E. Alvarez

**Affiliations:** 1 Department of Biomedical Sciences, Rocky Vista University College of Osteopathic Medicine, Englewood, CO 80112, USA; 2 Independent Researcher, Columbus, OH 43201, USA; 3 Department of Pediatrics, The Ohio State University College of Medicine, Columbus, OH 43210, USA; 4 Department of Veterinary Clinical Sciences, The Ohio State University College of Veterinary Medicine, Columbus, OH 43210, USA

**Keywords:** temperament, reactive personality, psychological stress, latent class analysis, C-BARQ, cancer risk, alleles, genetic, meta-analysis

## Abstract

**Simple Summary:**

It is widely accepted that long term mental stress increases the risk of cancer. However, it has been impossible to show this conclusively in any species due to the complexities of genetic variation and the difficulty of inducing or measuring levels of psychological stress. Here, we report evidence that heritable canine temperament that increases psychological stress in dogs is somehow linked to overall risk of multiple cancer types. We propose that studying this link in dogs would result in a new understanding of the relationship between psychological stress and risk of cancer and lead to new preventive and therapeutic veterinary medicine advances that could be tested in clinical trials in pet dogs.

**Abstract:**

Although there is evidence that psychological stress may be associated with increased cancer risk, the effect of stress on cancer risk is difficult to study, both in humans, due to socioeconomic factors, and in animal models, due to questionable biological relevance. Here, we test whether heritable canine temperament that increases psychological stress is associated with cancer risk. The study data are breed-specific averages of incidences of multiple cancer types and of temperament classes. The latter are derived from a latent class analysis of behavioral questionnaires completed by owners (C-BARQ). We thus classified the dogs according to whether they are calm vs. reactive within and across breeds. Using meta-analysis approaches, we modeled the risk of multiple cancer types in calm vs. reactive dogs. We adjusted for breed averages of body mass and lifespan, which are common confounders that impact cancer. Our study confirms that body size has a significant effect of on risk of multiple types of cancers in dogs and shows for the first time that temperament also has a moderate effect. These findings suggest dog models of heritable psychological stress are suitable for molecular epidemiological and translational studies on its effects on cancer risk.

## 1. Introduction

There is a longstanding and popular belief that mental stress in animals is positively associated with cancer risk. However, due to challenges to the evolutionary, epidemiological, and physiological approaches, it has been impossible to conclusively test this possibility. For instance, a shared ecological trait of long-lived invertebrate and vertebrate species is reduced risk of predation (and mortality in general [1]), and lifespan has been shown to be negatively associated with cancer risk (e.g., in sea urchins, Greenland sharks, Brandt’s bats, bowhead whales, and naked mole-rats). As predation risk can be reduced through diverse adaptations, such as large body size, flight, and group living, it is not straightforward to isolate the cancer effects of metabolism, mental stress, etc. [2].

Given the high levels of genetic and phenotypic heterogeneity, and the socioeconomic differences in humans, it is difficult to isolate and study the effects of stress and behavior that lead to cancer [3,4,5]. The large body of scientific studies has not yielded consistent evidence that specific behaviors, including psychological stress, can increase the risk of cancer and its development in humans [4,6,7,8,9]. The evidence for and against was recently reviewed by Falcinelli et al. [4], who examined 21 case-control or population studies on the effects of common types of acute and chronic psychological stress on cancer risk. Of these, 15 considered breast cancer alone, and only 3 tested for many or all cancer types; 13 of the 21 studies yielded positive results and 8 were negative (for breast cancer, 7 were positive and 9 were negative).

Despite the lack of clear evidence from prospective studies, and with a single study showing inconclusive findings [10], it is commonly believed that adverse effects of psychological stress on cancer risk and development are pervasive. This is reflected in the long history of investigation into the pathophysiology of stress related to all major aspects of cancer, an investigation that is currently active [4,11]. General pathways that are strongly affected by stress include the hypothalamic–pituitary axis and the sympathetic nervous system, which induce glucocorticoid and adrenaline/noradrenaline signaling. The duration of stress physiology can result in dramatically different effects. Chronic psychological stress has adverse consequences in multiple tissues through immune and inflammatory mechanisms. These can affect rates of bacterial/viral infections and DNA mutations, respectively. Many other cancer initiation mechanisms are also affected by stress. Moreover, psychopathology is associated with behaviors that are risk factors for cancer, such as smoking.

Since the optimal method for evaluating these factors in humans remains elusive and is complicated due to sampling and confounding issues, here we use a canine model to test for the association of psychological stress and cancer risk. Dogs are the most popular companion animal across the globe [12], and in many countries, such as the United States, they often receive highly sophisticated medical care [13]. In these societies, dogs commonly share environments with their human counterparts and thus are outstanding translational models. Additionally, due to the combination of extreme selection pressure for meeting breed standards and little to no selection for addressing health concerns, dogs have greatly reduced heterogeneity and polygenicity for diverse, complex traits [14]. Put another way, traits such as cancer risk and problematic behaviors tend to be explained by relatively very few variations with moderate to large effects, while in humans, these same traits can be explained by a great many variations with minute-to-small effects [15].

Like cancer traits, dog breed-specific behavioral profiles have been shown to have a genetic basis [16,17]. This has been carried out, in large part, through owner questionnaires mostly concerning problem behaviors such as different types of anxiety, fear, and aggression [18]. Breed averages of such temperament traits were recently used for genetic mapping [16,17]. Many of those mapped alleles were (i) shown to be associated with brain structure differences [19] and (ii) subsequently supported in a population sample of pedigree and mixed-breed dogs that included dogs with clinical behavioral diagnoses [20]. Dog temperament is associated with body size. As breed body size decreases, the risk of most problem behaviors—including reactive traits such as fear, anxiety, and aggression—increases [21,22]. This pattern can also be applied to lifespan since both dog size and lifespan are correlated [23]. Behavioral assessment batteries enable the broad classification of temperament profiles using latent class analysis (LCA) [22]. Rather than giving each breed an overall score for each temperament factor, this approach stratifies behavior into calm vs. fearful or aggressive classes and measures the percentage of each breed in each of these classes. Dog owners typically prefer dogs that are less prone to problematic behaviors, and this can be determined using C-BARQ, which evaluates problematic behavior, and LCA, which can stratify dogs into temperament profiles.

The combination of such canine cancer and temperament resources allows us to test whether problematic behavior that causes psychological stress in dogs is associated with cancer incidence. The relatively simple genetics of dogs presents the opportunity to quantify psychological stress across many breeds in a normalized way. Using this heritable predisposition for psychological stress obviates having to induce or measure environmental stress stimuli, as is typically the case in such studies on rodents and humans. While cancer effects could be confounded by breed-specific cancer risks, we mitigate that risk by using latent class analysis-derived temperament phenotypes, using an interbreed study design, and controlling for body size. Our findings have major implications for companion animal welfare and provide a powerful new insight into the links between stress and cancer risk in mammals in general.

## 2. Materials and Methods

### 2.1. Study Design

This is an exploratory study of breed stereotype association that combines multiple types of data related to stressful behavior, i.e., temperament profiles [22] derived from C-BARQ breed summaries [18], breed-specific genome-wide genotype data and derived genetic associations with behavioral traits relevant to stressful behavior (anxiety, fear, aggression, etc.) [17,19,20], and lifespan and body mass covariates. These data were modeled to detect an association between psychological stress and cancer incidence. The overall approach was to exploit breed stereotypes of behavior and cancer incidence to test for their association. A major advantage of studying domesticated animals with many breeds is the ability to measure association at loci which are fixed in some or most breeds. We mitigated possible sources of type 1 error caused by biased and internal population structures in the sample by using many breeds and multiple cohorts of genotype and phenotype data, as well as multiple types of cancer, and by running models with and without the inclusion of osteosarcoma data and breeds that are singletons across cancer types (e.g., see the effects of lymphoma risk in Fox Hounds and Irish Terriers in the Results section). Breed stereotype studies have provided a powerful discovery tool that has been used for many purposes in the past, such as morphological [24,25,26] and behavioral [16,17,20,25,26] genome-wide association studies.

### 2.2. Sources of Dog Breed-Specific Data for Cancer, Behavior, Lifespan, and Body Mass

A comprehensive data set was generated by combining multiple data from different sources, and these data included cancer incidence, behavior and associated genetic markers (temperament profiles from C-BARQ owner questionnaires, allele frequencies of genetic markers associated with behavioral traits), lifespan, and dog body mass. Data from a total of seven studies that included multi-breed cancer incidence data were included in this study. These studies presented data per breed for mast cell carcinoma [27], lymphoma [28,29], osteosarcoma [30,31,32], and melanoma [33]. A breakdown of the breeds represented in these studies is presented in Supplementary Table S1. The behavioral data, temperament group classification, and group distributions for each breed were sourced from LCA profile data generated from the C-BARQ questionnaire data of 57,454 dogs from 350 purebred and mixed-breed dogs [22]. The allele frequency data for the genetic markers associated with problematic behavior were previously reported [20] and were derived from a multi-breed dataset generated by Parker et al. [34]. The rationale for selecting this set of genetic markers for follow-up of our interbreed genetic mapping was that most were quasi-replicated across cohorts, while the others implicated genes prioritized for biological relevance (*ESR1*, *SHISA6*, *PRKG2*, *RASGEF1B*, and *IGF2BP2*) [17,19,20]. Breed lifespan and body mass data were obtained from the American Kennel Club (www.akc.org, accessed on 5 February 2023) and the Kennel Club (www.thekennelclub.org.uk accessed on 5 February 2023).

### 2.3. Statistical Analysis

Associations between cancer incidence and behavioral data were evaluated using generalized linear mixed models. All models were simultaneously assessed with lifespan and body mass as covariates. Since the sources of incidence data varied in sample size, the total sample size of each cancer incidence was introduced as a random effect adjustment in the model. Due to the nature of the data, residual distributions were assumed to be log normal. This assumption was based on residual panel assessments (residual plots, QQ plots, and residual histograms) of the models.

The analysis was run in two main modes: all breed data included, and singleton breeds removed. Singleton breeds are defined as those that are only represented in the study by a single assessment value in the comprehensive dataset. Removing these breeds reduces the risk of single breed observation bias. In addition, a set of models was run in the same modes, but excluding osteosarcoma. This was carried out because dog osteosarcoma is known to be closely associated with dog body size [31] (see Suppl. Text, [35]). All models were run using PROC GLIMMIX in SAS/STAT v.9.4 (SAS Institute Inc., Cary, NC, USA).

## 3. Results

We established previously that LCA categories distinguish between dogs that are more or less desirable to humans [22]. Dogs of higher desirability have temperament profiles that compile lower CBARQ scores. We thus use the LCA desirability classes to refer to the proportions of desired scores in behavioral profiles. In other words, behavioral desirability or calmness represents the absence, or the lowest level, of heritable predisposition to psychological stress, whereas the reactive classes are predisposed to moderate or high levels of psychological stress and are less desirable to dog owners. The evaluation of the models that included the LCA-generated temperament profiles and osteosarcoma data showed that cancer type and body size were consistently associated (Table 1, “Including Osteosarcoma”). In this first set of models, the effect of the cancer type dropped out once singleton breeds were removed. We speculate that this was due to rare breeds being included in such studies due to increased cancer incidence. The inclusion in lymphoma studies of Fox Hounds, which have an odds ratio of 34.37, and Irish Terriers, which have an odds ratio of 22.91 [29], are examples of this, and both are singleton breeds that were removed. After the removal of singleton breeds, only the effect of body size remained consistently associated.

When the temperament models were evaluated in the same modes (all breeds included and singleton breeds removed), but without the osteosarcoma data, only a few associations with temperament were detected (Table 1, “Not Including Osteosarcoma”). These were only found in comparisons in which the best, or lower stress, group was compared with all the others. The effect of body size was not relevant, except when evaluating osteosarcoma incidence. In all the significant cases, the risk of lymphoma was significantly higher in breeds within the best desirability group, i.e., those with the least stressed temperaments (*p* < 0.0001). This effect dropped once the single breeds were removed. An association between lifespan and cancer incidence was never detected when temperament was assessed.

We next evaluated the genetic markers associated with stressful behavior, as describes in the Materials and Methods section, and their association with cancer incidence. We observed in the first set of models, including osteosarcoma (Table 2), that three loci were positive (and that the most consistent effect was association with body size). Two loci on chromosome 10 (positions 2,431,382 for the first locus; 8,059,173, 8,397,696, and 8,454,499 for the second locus) and one marker on chromosome 32 (5,421,210) were associated with increased cancer risk. In contrast to the temperament profiles, the removal of singleton breeds did not change the overall pattern of association effects. The removal of singletons resulted in the loss of the chromosome 32 association but added a single association to the cancer type effect for the chr10:2,431,382 marker. In each model, as body size increased, the effect was one of increased incidence risk.

Next, we evaluated the same genetic markers associated with behavior, but we excluded the osteosarcoma data (Table 3). This resulted in a different pattern in which the body size effect was lost, but this replaced by a cancer type effect. In these models, variants on chromosomes 10 and 32 were again associated, as were new ones on chromosomes 1 and X. After the removal of singleton breeds, only the associations with variants in chromosomes 32 and X dropped out. Again, the risk of lymphoma was significantly higher (*p* < 0.0001). Lifespan was never found to be associated in any instance in which genetic markers were associated with stressful behavior and cancer incidence.

## 4. Discussion

Our results suggest that temperaments which increases psychological stress—including reactive states of fear, anxiety, and aggression—are positively associated with cancer incidence in dogs. Simply put, this is to say that a temperament that causes psychological stress is linked with a higher incidence of cancer in dogs. We first showed this through associations with behavioral profiles derived from a latent class analysis of high-powered data that included all common problem behaviors from 350 purebreds and mixed-breed dogs [22]. Among the positive findings, osteosarcoma and lymphoma had the strongest signal. For osteosarcoma, this is at least in part due to its association with larger body size, but more studies are necessary to understand cancer type effects. Given the great diversity of biological mechanisms involved in different cancer types, it would not be surprising if psychological stress greatly contributed to a subset of pathways (e.g., glucocorticoid and adrenergic signaling, immunological, and inflammatory) and thus cancer types [4,11]. Body size had a consistent effect associated with cancer incidence, but we were able to isolate or remove, as we explain below.

Dog breeds can have an increased or decreased germ line risk for diverse cancers and numbers of cancer types [36]. This has been established through epidemiological, genetic, and translational approaches that have identified oncogenic mechanisms and potential therapies, which in turn have led to veterinary clinical trials [35,37,38]. In a study relevant to the present approach and involving a cancer type included in this work, dogs were instrumental in identifying a cancer risk factor that was long suspected but difficult to determine in humans. Unlike in dogs, osteosarcoma is rare in humans. Due to its low incidence, its mostly sporadic nature, and socioeconomic and genetic confounding, it would be complicated to conclusively establish whether human osteosarcoma risk is associated with height [39]. However, in dogs it was possible to measure the effect of body mass on osteosarcoma risk [31] (see Suppl. Text, [35]). In this study, we mitigated breed effects due to body size, lifespan, and cancer predispositions by using many breeds and multiple cohorts of genotype and phenotype data, by controlling for body mass and lifespan in association models, and by separately running models with and without the inclusion of osteosarcoma data and singleton breeds across cancer types.

We next showed the effects of genetic markers associated with problem behaviors presumed to increase psychological stress [17,19,20]. Specifically, we found that genetic markers on chromosomes 1, 10, 32, and X were associated with increased cancer risk. Of these, the chromosome 10 and X markers were not only associated with canine behavior but also body size. Previous studies of individual genetic markers associated with canine body size have not detected associations with cancer risk, but it has been proposed that this is because these markers are fixed (homozygous) in many or most breeds [40]. A main advantage of our interbreed design is that it can detect associations of markers which are fixed in some or most breeds. These marker associations are important starting points for future studies. One possible use would be to identify breeds segregating both alleles to test for associations with cancer risk. Another possibility would be to conduct a focus study of these markers for canine cancers, such as osteosarcoma, with polygenic risk data [35], and this could feasibly include gene–gene and gene–environment study designs. Unlike the generally minute effect sizes of common individual variations associated with human psychopathology, these dog variations have moderate to large effect sizes and, thus, clinical utility [20]. Following further studies, it seems likely that genetic testing for these markers will be useful for dog selection and breeding, preventative veterinary medicine, and the development of therapies which would be validated in clinical trials in pet dogs.

The findings of this study have important implications for understanding the genetic and environmental factors that contribute to cancer incidence in dogs. It has long been assumed that stress physiology is likely to increase the risk of cancer and its development [4,11]. We favor that possibility, but we cannot rule out others, and the effects could be complex, multifactorial, and variable across cancer types. By identifying temperament profiles and genetic markers associated with increased cancer risk, this study provides valuable avenues through which the mechanisms involved can be studied. This will, in turn, produce insights into potential strategies for cancer prevention and early detection in dogs, and subsequently in other mammals. There are many ways to take advantage of the unique evolutionary history of dogs. Arguably, the most efficient approaches include studying specific large effect variations in individual breeds segregating both alleles. In this way, the overall genetic variation may be reduced one hundred-fold compared with the overall dog population. However, the functional locus can be tested for cancer risk in the homozygotes of both alleles and in heterozygotes (which show low, high, and intermediate effects, respectively). Once such genetic factors are established, they can, in turn, be used for measuring environmental effects and testing preventative interventions. Alternatively, higher-powered studies of mixed-breed dogs could be carried out using an admixture approach. Dogs are common (e.g., they number approximately 80 million in the USA, roughly half-and-half pedigree and mixed-breed), share the environments of their owners, frequently receive a high level of health care, and are generally considered family members. This presents a powerful opportunity to leverage observational and translational studies in pet dogs. Because psychological stress and cancer are widely understood to result in devastating morbidity and mortality in dogs and humans, dog owner interest in study participation is likely to be high.

It is important to note the limitations of this study. Due to the scarcity of canine epidemiological data, the study only examined a limited number of cancer types and did not evaluate other potential risk factors, such as environmental exposures. The future development of canine epidemiological data and cancer tissue repositories would have a great impact on this field of study [40,41]. In our study, we evaluated temperament, cancer type, dog size, and lifespan simultaneously in our models; all these factors are relevant to cancer risk, but they are also challenging because of confounding factors. Specific breeds are more likely to develop specific cancers, and they have defined weigh and lifespan ranges. To reduce this concern, we included as many breeds and cancers as the available data would permit. It is important to mention that even when dog size and lifespan are correlated, these effects did not display the same association in the models, suggesting that although there is some overlap, they have a component that is still distinct. Other confounders that may have a relevant association to cancer risk [42] cannot be evaluated from aggregate data. Additionally, rather than using phenotype and genotype data from the same dogs, the study relied on breed averages of owner-reported temperament data and the latent class analysis classes that were derived from these. It is unclear how robustly these measures reflect relative levels of psychological stress. However, it should be noted that we successfully performed diagnostic predictive modeling in a cohort of 397 dogs that included 122 dogs with a clinical behavioral diagnosis. Of the markers highlighted above, we correctly predicted diagnoses of anxiety for chr1, aggression for the second locus on chr10, and “any diagnosis” for the same chr10 locus and for chrX. It is also worth noting that the genetic mapping signal was compared using both the body size measurements of individual dogs and breed averages, and it was found that breed stereotypes had far greater power with the same number of dogs (in most cases, several to many orders of magnitude [15]).

## 5. Conclusions

The results of this study suggest that temperament associated with psychological stress is a factor in cancer incidence in dogs. This finding is important because it is difficult to conduct similar investigations in humans due to confounding socioeconomic and genetic factors, and it is difficult in rodents due to the requirement for inducible models of chronic stress. Further research is needed to fully understand the complex interplay between temperament and other factors, and to develop effective strategies for cancer prevention and treatment in dogs.

## Figures and Tables

**Table 1:** Associations between cancer risk and temperament profile, cancer type, body type, and lifespan (*p*-values). Associations including osteosarcoma are shown at the top, while associations excluding osteosarcoma are show at the bottom. The “desirability group comparison” corresponds to the pairwise comparison of groups with less or more desirable temperament profiles. The more desired groups are calmer, and thus are less prone to stress, while the less desired groups are more prone to stress. In this table, *n* corresponds to the individual breed observations across studies. Significant effects are labeled with an asterisk (*).

Including Osteosarcoma		All Breeds in Studies Included *n* = 157, 69 Breeds				Singleton Breeds in Studies Removed *n* = 129, 41 Breeds			
LCA Groups	Desirability Group Comparison	Temperament Effect	Cancer Type Effect	Body Size Effect	Lifespan Effect	Temperament Effect	Cancer Type Effect	Dog Size Effect	Lifespan Effect
2 groups	Best	0.7240	0.0387 *	3.3 × 10^−5^ *	0.1909	0.5032	0.1572	0.0006 *	0.4494
3 groups	Best	0.5881	0.0395 *	0.0001 *	0.1903	0.1230	0.1960	0.0074 *	0.5331
	Best and Mid	0.8579	0.0363 *	7.7 × 10^−6^ *	0.1992	0.7140	0.1154	0.0001 *	0.4885
4 groups	Best	0.6562	0.0390 *	0.0001 *	0.1938	0.2012	0.1788	0.0058 *	0.5537
	Best and 2nd Best	0.8427	0.0360 *	2.3 × 10^−5^ *	0.1918	0.9704	0.1282	0.0004 *	0.4828
	Best, 2nd Best, and 3rd Best	0.3482	0.0491 *	0.0001 *	0.2087	0.2213	0.2235	0.0015 *	0.5007
**Not Including Osteosarcoma**		**All Breeds in Studies Included *n* = 85, 50 Breeds**				**Singleton Breeds in Studies Removed *n* = 73, 38 Breeds**			
**LCA Groups**	**Desirability Group Comparison**	**Temperament Effect**	**Cancer Type Effect**	**Dog Size Effect**	**Lifespan Effect**	**Temperament Effect**	**Cancer Type Effect**	**Dog Size Effect**	**Lifespan Effect**
2 groups	Best	0.6386	0.0515	0.3050	0.7681	0.8597	0.1147	0.4323	0.8069
3 groups	Best	0.8875	0.0484 *	0.2702	0.7956	0.9293	0.1032	0.3890	0.7798
	Best and Mid	0.4984	0.0644	0.3877	0.8198	0.9472	0.1176	0.4190	0.7858
4 groups	Best	0.8372	0.0432 *	0.1985	0.8222	0.6024	0.0865	0.2641	0.7478
	Best and 2nd Best	0.5484	0.0376	0.1212	0.7744	0.3289	0.0763	0.1652	0.8462
	Best, 2nd Best, and 3rd Best	0.4957	0.0637	0.3493	0.8118	0.9056	0.1232	0.4190	0.7861

**Table 2:** Behaviorally relevant allele variant associations with cancer risk when osteosarcoma is included (*p*-values). Each of these assessments evaluates the joint effect of the specific allele, cancer type, dog size, and lifespan effects. In this table, *n* corresponds to the individual breed observations across studies. Significant effects are labeled with an asterisk (*).

		Including Osteosarcoma							
Position (CanFam3.1)	Allele	All Breeds in Studies Included *n* = 116, 42 Breeds				Singleton Breeds in Studies Removed *n* = 106, 32 Breeds			
		Allele Effect	Cancer Type Effect	Dog Size Effect	Lifespan Effect	Allele Effect	Cancer Type Effect	Dog Size Effect	Lifespan Effect
chr1:42035784	AA	0.1988	0.0933	0.0002 *	0.5433	0.2080	0.0934	0.0004 *	0.5674
	AB	0.2007	0.0771	0.0001 *	0.5249	0.2434	0.0787	0.0002 *	0.5774
	BB	0.4803	0.0976	0.0002 *	0.5781	0.4409	0.0971	0.0004 *	0.5725
chr1:93250465	AA	0.9606	0.0877	0.0002 *	0.5882	0.9578	0.0869	0.0003 *	0.5930
	AB	0.0676	0.6065	0.0001 *	0.6033	0.0985	0.0691	0.0002 *	0.6043
	BB	0.1831	0.0777	0.0002 *	0.6549	0.2297	0.0825	0.0003 *	0.6537
chr10:2431382	AA	0.0087 *	0.0688	0.0022 *	0.6119	0.0120 *	0.0546	0.0048 *	0.5065
	AB	0.0329 *	0.0592	0.0004 *	0.9178	0.0374 *	0.0479 *	0.0010 *	0.9519
	BB	0.0106 *	0.0963	0.0050 *	0.4406	0.0165 *	0.0794	0.0082 *	0.4186
chr10:7996770	AA	0.6759	0.0843	0.0001 *	0.5509	0.7060	0.0828	0.0002 *	0.5650
	AB	0.2428	0.0979	0.0001 *	0.4118	0.2426	0.0920	0.0001 *	0.4271
	BB	0.5624	0.1010	0.0001 *	0.5411	0.5442	0.1012	0.0002 *	0.5347
chr10:8059173	AA	0.0736	0.0780	0.0001 *	0.3386	0.1708	0.0945	0.0002 *	0.4248
	AB	0.3650	0.0907	0.0001 *	0.6090	0.3103	0.0775	0.0003 *	0.6358
	BB	0.0200 *	0.0831	0.0001 *	0.3110	0.0424 *	0.0879	0.0002 *	0.4023
chr10:8397696	AA	0.0769	0.0670	0.0001 *	0.5891	0.0962	0.0777	0.0002 *	0.6191
	AB	0.0461 *	0.0613	0.0001 *	0.5335	0.0351 *	0.0658	0.0002 *	0.5455
	BB	0.4251	0.0826	0.0001 *	0.6054	0.6085	0.0868	0.0002 *	0.6104
chr10:8454499	AA	0.0502	0.1263	0.0001 *	0.6279	0.0665	0.1237	0.0001 *	0.5766
	AB	0.0483 *	0.1547	0.0001 *	0.4278	0.0253 *	0.1500	0.0003 *	0.5197
	BB	0.0238 *	0.1724	0.0001 *	0.4840	0.0189 *	0.1648	0.0001 *	0.5363
chr13:8391652	AA	0.3068	0.0975	0.0001 *	0.7448	0.2160	0.0835	0.0004 *	0.9149
	AB	0.9736	0.0864	0.0001 *	0.5872	0.7953	0.0829	0.0002 *	0.6304
	BB	0.5316	0.0956	0.0001 *	0.5957	0.5964	0.0950	0.0003 *	0.6069
chr15:41232547	AA	0.7816	0.0832	0.0001 *	0.5548	0.8694	0.0849	0.0003 *	0.5685
	AB	0.3195	0.0676	0.0001 *	0.5826	0.3346	0.0668	0.0002 *	0.5523
	BB	0.4710	0.0920	0.0006 *	0.7562	0.3684	0.0977	0.0013 *	0.8070
chr15:41250986	AA	0.8940	0.0965	0.0001 *	0.5733	0.6173	0.0938	0.0002 *	0.5397
	AB	0.9273	0.1026	0.0001 *	0.5820	0.6526	0.1033	0.0002 *	0.5822
	BB	0.9176	0.0863	0.0001 *	0.5726	0.7948	0.0837	0.0002 *	0.5560
chr18:20310833	AA	0.1201	0.1032	0.0002 *	0.8017	0.4098	0.0886	0.0004 *	0.7369
	AB	0.4390	0.0786	0.0001 *	0.6488	0.8024	0.0838	0.0002 *	0.6016
	BB	0.2140	0.1146	0.0002 *	0.7216	0.4544	0.0921	0.0004 *	0.7241
chr24:23196435	AA	0.7597	0.0826	0.0002 *	0.6839	0.5325	0.0767	0.0003 *	0.7973
	AB	0.5350	0.0774	0.0001 *	0.5997	0.5110	0.0783	0.0002 *	0.6128
	BB	0.8905	0.0891	0.0001 *	0.5745	0.8207	0.0834	0.0003 *	0.6985
chr24:23398090	AA	0.8298	0.0858	0.0003 *	0.6270	0.9589	0.0851	0.0004 *	0.5849
	AB	0.7229	0.0875	0.0001 *	0.5353	0.5743	0.0870	0.0002 *	0.5051
	BB	0.2195	0.0924	0.0005 *	0.7307	0.3146	0.0960	0.0007 *	0.6514
chr32:5421210	AA	0.0476 *	0.0647	0.0003 *	0.9080	0.0828	0.0866	0.0006 *	0.8405
	AB	0.1234	0.0807	0.0004 *	0.9499	0.1205	0.1078	0.0012 *	0.9518
	BB	0.0800	0.0601	0.0001 *	0.5929	0.1947	0.0703	0.0002 *	0.5682
chr34:18559537	AA	0.3907	0.0802	0.0001 *	0.5525	0.2008	0.0862	0.0002 *	0.5776
	AB	0.3705	0.0783	0.0001 *	0.5353	0.1884	0.0840	0.0002 *	0.5435
	BB	0.5692	0.0865	0.0002 *	0.5993	0.3723	0.0907	0.0004 *	0.6554
chrX:101646292	AA	0.1769	0.0748	0.0001 *	0.3733	0.1566	0.0803	0.0002 *	0.3681
	AB	0.6138	0.0857	0.0001 *	0.6033	0.8379	0.0867	0.0003 *	0.5884
	BB	0.1154	0.0735	0.0001 *	0.3702	0.1386	0.0854	0.0001 *	0.3758
chrX:102553876	AA	0.9228	0.0948	0.0002 *	0.5732	0.7385	0.0910	0.0003 *	0.5425
	AB	0.6621	0.0806	0.0002 *	0.6122	0.9387	0.0850	0.0003 *	0.5950
	BB	0.0781	0.2528	<0.0001 *	0.4056	0.0764	0.1856	<0.0001 *	0.3674

**Table 3:** Behaviorally relevant allele variant associations with cancer risk when osteosarcoma is excluded (*p*-values). Each of these assessments evaluates the joint effect of the specific allele, cancer type, dog size, and lifespan effects. In this table, *n* corresponds to the individual breed observations across studies. Significant effects are labeled with an asterisk (*).

		Not Including Osteosarcoma							
Position (CanFam3.1)	Allele	All Breeds in Studies Included *n* = 88, 35 Breeds				Singleton Breeds in Studies Removed *n* = 74, 30 Breeds			
		Allele Effect	Cancer Type Effect	Dog Size Effect	Lifespan Effect	Allele Effect	Cancer Type Effect	Dog Size Effect	Lifespan Effect
chr1:42035784	AA	0.9614	0.0304 *	0.1957	0.8151	0.7807	0.0371 *	0.3545	0.9966
	AB	0.4069	0.0279 *	0.2146	0.8440	0.2115	0.0310 *	0.4202	0.9253
	BB	0.3668	0.0232 *	0.1578	0.9201	0.3475	0.0262 *	0.2582	0.8987
chr1:93250465	AA	0.5405	0.0346 *	0.2364	0.8897	0.5090	0.0410 *	0.3907	0.9157
	AB	0.0159 *	0.0115 *	0.2080	0.9006	0.0247 *	0.0198 *	0.3986	0.8873
	BB	0.0514 *	0.0208 *	0.2627	0.9693	0.0652	0.0302 *	0.4548	0.8302
chr10:2431382	AA	0.0009 *	0.0144 *	0.7134	0.2411	0.0009 *	0.0104 *	0.9130	0.1177
	AB	0.0041 *	0.0113 *	0.3886	0.6198	0.0020 *	0.0068 *	0.7950	0.2991
	BB	0.0029 *	0.0306 *	0.8166	0.2021	0.0050 *	0.0260 *	0.9996	0.1564
chr10:7996770	AA	0.6705	0.0301 *	0.2020	0.8318	0.6814	0.0365 *	0.3255	0.9900
	AB	0.6786	0.0279 *	0.2072	0.8753	0.6660	0.0331 *	0.3286	0.9453
	BB	0.8310	0.0344 *	0.1904	0.7956	0.8642	0.0401 *	0.3204	0.9836
chr10:8059173	AA	0.0953	0.0260 *	0.1718	0.5862	0.1433	0.0404 *	0.3469	0.8050
	AB	0.2891	0.0311 *	0.2048	0.8703	0.2016	0.0280 *	0.3586	0.9111
	BB	0.0171 *	0.0256 *	0.1812	0.5515	0.0165 *	0.0328 *	0.4171	0.7676
chr10:8397696	AA	0.0170 *	0.0166 *	0.1383	0.8919	0.0369 *	0.0269 *	0.2735	0.9248
	AB	0.0099 *	0.0128 *	0.1213	0.7306	0.0113 *	0.0180 *	0.2334	0.9045
	BB	0.1922	0.0271 *	0.1785	0.9182	0.3564	0.0364 *	0.3177	0.9201
chr10:8454499	AA	0.0093 *	0.0551	0.0783	0.7428	0.0155 *	0.0611	0.1576	0.9486
	AB	0.2677	0.0575	0.1956	0.7517	0.1653	0.0675	0.3323	0.9434
	BB	0.0441 *	0.0774	0.1351	0.7117	0.0358 *	0.0831	0.2352	0.9209
chr13:8391652	AA	0.1529	0.0361 *	0.1759	0.9763	0.0966	0.0297 *	0.3434	0.6313
	AB	0.8148	0.0292 *	0.1889	0.8511	0.4341	0.0269 *	0.3062	0.8642
	BB	0.4456	0.0364 *	0.1894	0.8185	0.6732	0.0405 *	0.3297	0.9877
chr15:41232547	AA	0.5398	0.0256 *	0.1561	0.7301	0.7833	0.0340 *	0.3029	0.9596
	AB	0.2113	0.0186 *	0.1553	0.7726	0.3720	0.0260 *	0.2803	0.9680
	BB	0.6878	0.0314 *	0.2856	0.9316	0.5230	0.0395 *	0.4903	0.8266
chr15:41250986	AA	0.7143	0.0425 *	0.1871	0.7823	0.5093	0.0422 *	0.3070	0.9427
	AB	0.9493	0.0432 *	0.1901	0.8168	0.6441	0.0496 *	0.3013	0.9895
	BB	0.6454	0.0301 *	0.1954	0.7562	0.6299	0.0333 *	0.3277	0.9361
chr18:20310833	AA	0.4142	0.0363 *	0.2337	0.9995	0.7724	0.0360 *	0.3487	0.9325
	AB	0.7586	0.0288 *	0.1895	0.8365	0.9189	0.0350 *	0.3215	0.9916
	BB	0.4729	0.0426 *	0.2545	0.9940	0.8402	0.0369 *	0.3536	0.9459
chr24:23196435	AA	0.1783	0.0178 *	0.2074	0.8332	0.1178	0.0208 *	0.3532	0.6150
	AB	0.5661	0.0261 *	0.1850	0.8338	0.4624	0.0302 *	0.3132	0.9760
	BB	0.1946	0.0209 *	0.2270	0.7706	0.1507	0.0241 *	0.3769	0.5745
chr24:23398090	AA	0.2807	0.0290 *	0.1293	0.6862	0.3243	0.0333 *	0.2182	0.8575
	AB	0.2244	0.0311 *	0.1282	0.6639	0.2542	0.0365 *	0.2128	0.8292
	BB	0.8011	0.0292 *	0.1825	0.8041	0.8821	0.0348 *	0.3164	0.9940
chr32:5421210	AA	0.0510	0.0207 *	0.2459	0.8443	0.0631	0.0345 *	0.4719	0.6546
	AB	0.3732	0.0297 *	0.2447	0.9410	0.2329	0.0471 *	0.5038	0.6563
	BB	0.0144 *	0.0134 *	0.1604	0.8044	0.0531	0.0214 *	0.2964	0.9951
chr34:18559537	AA	0.3730	0.0263 *	0.2344	0.8631	0.3167	0.0351 *	0.3692	0.9665
	AB	0.3328	0.0251 *	0.2224	0.8444	0.2754	0.0340 *	0.3466	0.9948
	BB	0.6352	0.0299 *	0.2384	0.8722	0.5842	0.0370 *	0.3877	0.9389
chrX:101646292	AA	0.1307	0.0228 *	0.1232	0.4532	0.0877	0.0309 *	0.2608	0.5636
	AB	0.2748	0.0286 *	0.1501	0.9254	0.6502	0.0365 *	0.2863	0.9596
	BB	0.0328 *	0.0191 *	0.0758	0.3922	0.0551	0.0333 *	0.1809	0.5846
chrX:102553876	AA	0.3153	0.0498 *	0.1325	0.6662	0.1410	0.0460 *	0.1485	0.6949
	AB	0.6056	0.0351 *	0.1676	0.7533	0.2291	0.0341 *	0.2026	0.8118
	BB	0.0961	0.1351	0.0948	0.6566	0.1563	0.1055	0.1362	0.7568

## Data Availability

Data are available on reasonable request to the corresponding author.

## Supplementary Materials

The following supporting information can be downloaded at: https://www.mdpi.com/article/10.3390/ani13111869/s1, References [27,28,29,30,31,32,33] are cited in the supplementary materials.
