# Supplementary material for: Psychological Stress Is Associated with Increased Cancer Risk in Dogs
Source: Animals (Basel). 2023 Jun 3;13(11):1869. doi: 10.3390/ani13111869 (PMC10251977; doi:10.3390/ani13111869)
Supplement: Supplementary file 1 [file animals-13-01869-s001.zip › animals-2344235-supplementary.pdf]

Supplementary material

Breeds included	Mast Cell (Śmiech et al. 2018) N=2,763	Lymphoma (Edwards et al. 2003) N=83,589	Lymphoma (Yau et al. 2017) N=7,067	Osteosarcoma (Edmunds et al. 2021) N=444,473	Osteosarcoma (Ru, Terracini, and Glickman 1998) N=4,364	Osteosarcoma (Sapierzyński and Czopowicz 2017) N=2,604	Melanoma (Gillard et al. 2014) N=15,805
Akita				■			
Alaskan Malamute			■	■			
American Bulldog				■			
American Staffordshire Terrier	■		■			■	
Australian Cattle Dog			■				
Beagle			■	■			
Beauceron							■
Bernese Mountain Dog	■		■				
Bichon Frise				■			
Border Collie		■	■	■			
Border Terrier				■			
Boxer	■	■	■	■		■	
Bulldog		■		■			
Bullmastiff		■	■	■			
Bull Terrier	■						
Cairn Terrier				■		■	
Cane Corso						■	
Caucasian Shepherd Dog	■					■	
Cavalier King Charles Spaniel		■	■	■			
English Cocker Spaniel	■	■	■	■	■		
Collie				■	■	■	
Dachshund	■				■		
Dalmatian		■		■			
Doberman Pinscher	■	■	■	■	■	■	
Dogue de Bordeaux				■			
English Springer Spaniel		■	■	■			
Fox Hound			■				
Fox Terrier			■	■			
French Bulldog	■			■			
German Shepherd Dog	■	■	■	■	■	■	
Giant Schnauzer			■			■	
Golden Retriever	■	■	■	■	■	■	

Great Dane							
Greyhound							
Hungarian Vizla							
Irish Setter							
Irish Terrier							
Irish Wolfhound							
Jack Russell Terrier							
Keeshond							
Kelpie							
Labradoodle							
Labrador Retriever							
Lurcher							
Maltese							
Miniature Fox Terrier							
Miniature Poodle							
Miniature Schnauzer							
Old English Shepherd Dog							
German Shorthaired Pointer							
Polish Tatra Sheepdog							
Pug							
Rhodesian Ridgeback							
Rottweiler							
Standard Schnauzer							
Scottish Terrier							
Shar-Pei							
Shetland Shepherd Dog							
Shih Tzu							
Siberian Husky							
Silky Terrier							
Staffordshire Bull Terrier							
Saint Bernard							
Standard Poodle							
Tibetan Terrier							
Weimaraner							
West Highland Terrier							
Whippet							
Yorkshire Terrier							

References

Edmunds, Grace L., Matthew J. Smalley, Sam Beck, Rachel J. Errington, Sara Gould, Helen Winter, Dave C. Brodbelt, and Dan G. O’Neill. 2021. “Dog Breeds and Body Conformations with Predisposition to Osteosarcoma in the UK: A Case-Control Study.” *Canine Medicine and Genetics* 8 (1): 2. https://doi.org/10.1186/s40575-021-00100-7.

Edwards, D S, W E Henley, E F Harding, J M Dobson, and J L N Wood. 2003. “Breed Incidence of Lymphoma in a UK Population of Insured Dogs.” *Veterinary and Comparative Oncology* 1 (4): 200–206. https://doi.org/https://doi.org/10.1111/j.1476-5810.2003.00025.x.

Gillard, Marc, Edouard Cadieu, Clotilde De Brito, Jérôme Abadie, Béatrice Vergier, Patrick Devauchelle, Frédérique Degorce, et al. 2014. “Naturally Occurring Melanomas in Dogs as Models for Non-UV Pathways of Human Melanomas.” *Pigment Cell & Melanoma Research* 27 (1): 90–102. https://doi.org/10.1111/pcmr.12170.

Ru, G, B Terracini, and L T Glickman. 1998. “Host Related Risk Factors for Canine Osteosarcoma.” *The Veterinary Journal* 156 (1): 31–39. https://doi.org/https://doi.org/10.1016/S1090-0233(98)80059-2.

Sapierzyński, R., and M. Czopowicz. 2017. “The Animal-Dependent Risk Factors in Canine Osteosarcomas.” *Polish Journal of Veterinary Sciences* 20 (2): 293–98. https://doi.org/10.1515/pjvs-2017-0035.

Śmiech, Anna, Brygida Ślaska, Wojciech Łopuszyński, Agnieszka Jasik, Diana Bochyńska, and Roman Dąbrowski. 2018. “Epidemiological Assessment of the Risk of Canine Mast Cell Tumours Based on the Kiupel Two-Grade Malignancy Classification.” *Acta Veterinaria Scandinavica* 60 (1): 70. https://doi.org/10.1186/s13028-018-0424-2.

Yau, P P Y, N K Dhand, P C Thomson, and R M Taylor. 2017. “Retrospective Study on the Occurrence of Canine Lymphoma and Associated Breed Risks in a Population of Dogs in NSW (2001–2009).” *Australian Veterinary Journal* 95 (5): 149–55. https://doi.org/https://doi.org/10.1111/avj.12576.
